# There is no such thing as a Ministry of Truth and why it is important to challenge conventional “wisdom” - A personal view

**DOI:** 10.1016/j.nmni.2023.101155

**Published:** 2023-06-22

**Authors:** Philippe Brouqui, Michel Drancourt, Didier Raoult

**Affiliations:** Aix Marseille Université, IHU-Méditerranée Infection, Marseille, France

George Orwell described, in his highly topical work “1984”, a *Ministry of Truth* that imposed itself on everyone justify the censorship of anything that was not compatible with the doctrine of this Ministry [1]. A good example of this type of censorship is what was officially claimed by YouTube for the use of Hydroxychloroquine in the treatment of COVID (Fig. 1).

**Fig. 1:**
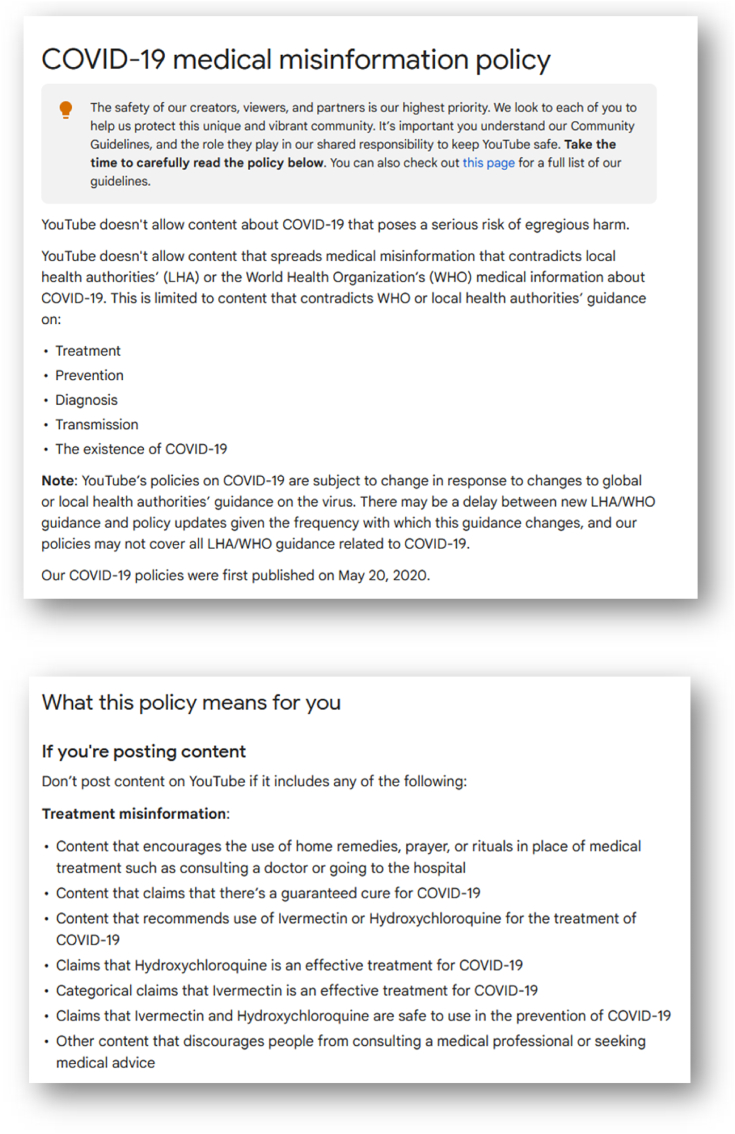
Screen capture of the Youtube policies on COVID-19. Available at https://support.google.com/youtube/answer/9891785?hl=en.

To justify censorship, some official statements, such as the FDA's tweet that “if you are not a horse or cow, do not take ivermectin, which is an animal medicine” are false information. This is obviously not ignorance on the part of the FDA, since it is well known that ivermectin is one of the most widely used human antiparasitic drugs in the world, but rather pure propaganda that legitimizes censorship over the use of ivermectin.

Censorship applies not only in social networks and in the usual media, but also apply in scientific journals. The first article on HCQ efficacy came from China, and we published preliminary results showing a faster decrease of the viral load in patients treated with HCQ combined with azithromycin [2]. Since then, 476 studies testing HCQ, have been released. Only studies reporting a lack of efficacy were published in highly impacted journals. However, these studies conclusions were not supported by the data. The data support a notable difference in favor to HCQ, but it was erroneously concluded that this was not, because this difference was not significant. A scientific impartial conclusion of these studies should have been that HCQ showed positive efficacy, but the inconclusive (not significant) results suggest that the studies might be underpowered.

While HCQ, a very old worldwide prescribed molecule, was reported to be very safe, its toxicity of was requestioned. However once again this was a pure propaganda. The Lancet publication (now retracted) could only be a fake, as the risk factors presented in the patients were the same on all continents and they enrolled in Australia more patients than the total cases reported in this country. The RECOVERY study reported a 0.4% excess of cardiac death, but this study used a massive and toxic dosage of 2400 mg of HCQ the first 24 hours [3]. Nevertheless, these studies then served to spread the misconception that HCQ presented a risk of cardiac toxicity, while at usual dosages it was used for decades in millions of people. This served as a scientific proof to force policy makers to stop ongoing studies leading to publish inconclusive underpowered studies. Thereafter, censorship was quickly imposed on all the works that reported a positive effect of HCQ, contrasting with the considerable amount of scientific published data reported. This suggest a lack of “official truth” on HCQ as there is no Ministry of truth on this topic.

Our Ministry of health claimed that general vaccination would prevent the contamination of subjects at risk. Since then, it has been shown that no control of the disease, worldwide, was associated with vaccination. This is very easily seen by looking at the Johns Hopkins web site. The epidemic in South Korea, UK, Israel, and Iceland followed the vaccination campaigns. Pfizer senior executive Janine Small revealed the vaccine was not tested for transmission before it entered the market due to the speed other drug companies were developing their products [4]. We issued a call for caution on the efficacy of vaccination to curtail the transmission and spread of the disease and called for a pragmatic assessment of the risk-benefit ratio of vaccination. Nevertheless, all attempts to mitigate the enthusiasm about the control of the disease by vaccination have also been censored by social networks, the mainstream press, and the scientific press. These censorship includes retraction of papers pointing out vaccine safety problems, negative publicity, difficulty to obtain research funding, call for dismissal, summonses of official hearings, suspension of medical licenses, and self-censorship [5].

Cyber-harassment is another tactic to discredit highly cited researcher that bring scientific controversy during COVID-19. We were the target of a cyber harassment campaign on “Pubpeer”, by anonymous individuals presenting themselves as specialists in scientific fraud, and who have undertaken to examine all the studies of the members of IHU. Pubpeer has commented on more than 350 articles in which at least one of the members of the IHU is referred as an author. Most of these comments are absurd, but some accuse IHU members of neo-colonial science, which is legally reprehensible. It goes without saying that anonymous comments are problematic insofar as there is no information on the scientific background of the commentators. As such, this goes far beyond the scope of peer review and becomes cyber harassment in the sole purpose to undermine the reputation of the IHU.

Anyway, partially based on these comments, an eight-month investigation was conducted at the IHU by the general inspection of public health and research ministry (IGAS-IGESR) and the French drug agency (ANSM). Those investigations led to the examination of 30,000 pages of documents and 700 hours of interviews. During those investigations, several hundreds of articles were reviewed. The outcome of the inspection was that there was disagreement between the inspectors and us on only two studies. Not fully satisfied of their impact on the IHU, individuals from that organisation also wrote to 90 journal editors to inform them of possible scientific fraud. Some journal editors, who were alerted by Pubpeer have flagged the cited articles with “expression of concerns” without giving us any possibility to defend ourselves and express that they will not publish our papers anymore. PLOS decided to flag an “expression of concern” on 49 articles published whenever one author belongs to the IHU. Our eight highly cited researchers were not evaluated in 2022 by Clarivate® because of this and disappeared from the list. This cyber harassment might have a considerable impact on the research output of the most productive centre in infectious diseases in France, by discrediting the IHU on the quality and integrity of the research carried out over all these years.

Conversely, a major ethical problem is conflict of interest, which is currently still neglected by most journals at a time when, on the contrary, it is responsible for scientific misconduct. The amount of money received from Gilead Sciences by physician was correlated with their public opposition to the use of HCQ in France. Self-declaration is not sufficient and active research through the available data banks, such as, for example EurosForDocs, should be systematically performed by responsible journal ethics teams. This naturally also applies to whistle-blowers.

The campaign coordinated by Pubpeer raises the question of the goal of these platforms and the role played by the media in discrediting high quality, renowned scientists who have no links to the pharmaceutical industries and who did not benefit from the pandemic. Science is a debate with rules. When someone disagrees with the scientific content of an article, comments should be addressed to the journal and be peer reviewed by recognised experts without any conflict of interest, in such a way as to preserve independent and constructive debate and enrich the quality of science. Most of the greatest scientific and medical advances in history were achieved by researchers who challenged the “Ministry of Truth”.

## Fundings

This work has benefited from State aid managed by the National Research Agency under the “Investments for the Future” program bearing the reference Méditerranée Infection 10-IAHU-03.

## Declaration of competing interest

None.
