# Supplementary material for: There is no such thing as a Ministry of Truth and why it is important to challenge conventional “wisdom” - A personal view
Source: New Microbes New Infect. 2023 Jun 22;54:101155. doi: 10.1016/j.nmni.2023.101155 (PMC10320372; doi:10.1016/j.nmni.2023.101155)
Supplement: Multimedia component 1 [file mmc1.docx]

**Online supplemental data**

There is no such thing as a Ministry of Truth and why it is important to challenge conventional “wisdom” - a personal view.

**Authors :** Philippe BROUQUI, Michel DRANCOURT & Didier RAOULT

**Supplement literature**

1. George Orwell. Nineteen Eighty-Four (1984). Secker & Warburg; 1949.
2. Liester, Mitchell B. The Suppression of Dissent During the COVID-19 Pandemic. Social Epistemology Review and Reply Collective [Internet]. 2022 Apr 21 [cited 2022 Dec 7]; Available from: https://social-epistemology.com/2022/04/21/the-suppression-of-dissent-during-the-covid-19-pandemic-mitchell-b-liester/
3. Niemiec E. COVID-19 and misinformation: Is censorship of social media a remedy to the spread of medical misinformation? EMBO Rep. 2020 Nov 5;21(11):e51420.
4. Shir-Raz Y, Elisha E, Martin B, Ronel N, Guetzkow J. Censorship and Suppression of Covid-19 Heterodoxy: Tactics and Counter-Tactics. Minerva. 2022 Nov 1;1–27.
5. Elisha E, Guetzkow J, Shir-Raz Y, Ronel N. Suppressing Scientific Discourse on Vaccines? Self-perceptions of researchers and practitioners. HEC Forum. 2022 May 19;1–19.
6. Ashour DS. Ivermectin: From theory to clinical application. Int J Antimicrob Agents. 2019 Aug;54(2):134–42.
7. Ivermectin | COVID-19 Treatment Guidelines [Internet]. [cited 2022 Dec 7]. Available from: https://www.covid19treatmentguidelines.nih.gov/therapies/miscellaneous-drugs/ivermectin/
8. Sanchezruiz WL, Nuzum DS, Kouzi SA. Oral ivermectin for the treatment of head lice infestation. Am J Health Syst Pharm. 2018 Jul 1;75(13):937–43.
9. Gautret P, Lagier JC, Parola P, Hoang VT, Meddeb L, Mailhe M, et al. HCQ and azithromycin as a treatment of COVID-19: results of an open-label non-randomized clinical trial. International Journal of Antimicrobial Agents. 2020 Jul;56(1):105949.
10. TOUT COMPRENDRE - Didier Raoult accusé de fraude par ses équipes sur l’HCQ [Internet]. [cited 2022 Dec 13]. Available from: https://www.bfmtv.com/police-justice/tout-comprendre-didier-raoult-accuse-de-fraude-par-ses-equipes-sur-l-HCQ_AV-202111200131.html
11. Mehra MR, Desai SS, Ruschitzka F, Patel AN. RETRACTED: HCQ or chloroquine with or without a macrolide for treatment of COVID-19: a multinational registry analysis. Lancet. 2020 May 22;
12. Lane JCE, Weaver J, Kostka K, Duarte-Salles T, Abrahao MTF, Alghoul H, et al. Risk of HCQ alone and in combination with azithromycin in the treatment of rheumatoid arthritis: a multinational, retrospective study. Lancet Rheumatol. 2020 Nov;2(11):e698–711.
13. Lagier JC, Million M, Gautret P, Colson P, Cortaredona S, Giraud-Gatineau A, et al. Outcomes of 3,737 COVID-19 patients treated with HCQ/azithromycin and other regimens in Marseille, France: A retrospective analysis. Travel Med Infect Dis. 2020 Aug;36:101791.
14. Raoult D. Lancet gate: a matter of fact or a matter of concern. New Microbes New Infect. 2020 Sep 22;38:100758.
15. Dubée V, Roy PM, Vielle B, Parot-Schinkel E, Blanchet O, Darsonval A, et al. HCQ in mild-to-moderate coronavirus disease 2019: a placebo-controlled double blind trial. Clinical Microbiology and Infection [Internet]. 2021 Mar 31 [cited 2021 Jun 2];0(0). Available from: https://www.clinicalmicrobiologyandinfection.com/article/S1198-743X(21)00140-3/abstract
16. RECOVERY Collaborative Group, Horby P, Mafham M, Linsell L, Bell JL, Staplin N, et al. Effect of HCQ in Hospitalized Patients with Covid-19. N Engl J Med. 2020 Nov 19;383(21):2030–40.
17. Axfors C, Schmitt AM, Janiaud P, Van’t Hooft J, Abd-Elsalam S, Abdo EF, et al. Mortality outcomes with HCQ and chloroquine in COVID-19 from an international collaborative meta-analysis of randomized trials. Nat Commun. 2021 Apr 15;12(1):2349.
18. COVID-19 early treatment: real-time analysis of 2,337 studies [Internet]. [cited 2022 Dec 12]. Available from: https://c19early.org/
19. “Le vaccin est 100% efficace contre le confinement” (Véran) | AFP Extrait [Internet]. 2021 [cited 2022 Dec 12]. Available from: https://www.youtube.com/watch?v=UmwwNPnbLNg
20. COVID-19 Map - Johns Hopkins Coronavirus Resource Center [Internet]. [cited 2022 Dec 12]. Available from: https://coronavirus.jhu.edu/map.html
21. Pfizer vaccine not tested for transmission due to ‘speed of science’ [Internet]. [cited 2022 Dec 12]. Available from: https://www.hitc.com/en-gb/2022/10/12/pfizer-vaccine-not-tested-for-transmission-due-to-speed-of-science/
22. Brouqui P, Boudjema S, Soto Aladro A, Chabrière E, Florea O, Nguyen H, et al. New Approaches to Prevent Healthcare-Associated Infection. Clin Infect Dis. 2017 Aug 15;65(suppl_1):S50–4.
23. Brouqui P, Drancourt M, Raoult D, on behalf of the IHU Task Force. COVID-19 Management at IHU Méditerranée Infection: A One-Year Experience. Journal of Clinical Medicine. 2021 Jan;10(13):2881.
24. La Scola B, Le Bideau M, Andreani J, Hoang VT, Grimaldier C, Colson P, et al. Viral RNA load as determined by cell culture as a management tool for discharge of SARS-CoV-2 patients from infectious disease wards. Eur J Clin Microbiol Infect Dis. 2020 Jun;39(6):1059–61.
25. Andreani J, Le Bideau M, Duflot I, Jardot P, Rolland C, Boxberger M, et al. In vitro testing of combined HCQ and azithromycin on SARS-CoV-2 shows synergistic effect. Microb Pathog. 2020 Aug;145:104228.
26. Colson P, Fournier PE, Chaudet H, Delerce J, Giraud-Gatineau A, Houhamdi L, et al. Analysis of SARS-CoV-2 Variants From 24,181 Patients Exemplifies the Role of Globalization and Zoonosis in Pandemics. Frontiers in Microbiology [Internet]. 2022 [cited 2022 Nov 23];12. Available from: https://www.frontiersin.org/articles/10.3389/fmicb.2021.786233
27. Million M, Lagier JC, Tissot-Dupont H, Ravaux I, Dhiver C, Tomei C, et al. Early combination therapy with HCQ and azithromycin reduces mortality in 10,429 COVID-19 outpatients. Rev Cardiovasc Med. 2021 Sep 24;22(3):1063–72.
28. Lagier JC, Million M, Cortaredona S, Delorme L, Colson P, Fournier PE, et al. Outcomes of 2111 COVID-19 Hospitalized Patients Treated with HCQ/Azithromycin and Other Regimens in Marseille, France, 2020: A Monocentric Retrospective Analysis. Ther Clin Risk Manag. 2022;18:603–17.
29. Professeur Didier Raoult. Au dela de l’affaire de la choroquine [Internet]. Michel Lafon. Michel Lafon; 2021 [cited 2022 Nov 23]. Available from: https://www.amazon.fr/Au-del%C3%A0-laffaire-chloroquine-Didier-Raoult/dp/2749949033/ref=sr_1_18?adgrpid=1351300825091059&hvadid=84456759752602&hvbmt=bp&hvdev=c&hvlocphy=126943&hvnetw=o&hvqmt=p&hvtargid=kwd-84456889646595%3Aloc-66&hydadcr=9753_2235779&keywords=livre+sur+raoult&qid=1669200065&qu=eyJxc2MiOiIwLjAwIiwicXNhIjoiMC4wMCIsInFzcCI6IjAuMDAifQ%3D%3D&sr=8-18
30. Retraction Watch. Paper co-authored by sleuth Elisabeth Bik marked with expression of concern. [cited 2022 Nov 23]; Available from: https://retractionwatch.com/2022/10/20/paper-co-authored-by-sleuth-elisabeth-bik-marked-with-expression-of-concern/
31. Elisabeth Bik, "la déceleuse de fraudes scientifiques" qui critique constamment Didier Raoult, est-elle au-dessus de tout soupçon ? | FranceSoir [Internet]. [cited 2022 Dec 12]. Available from: https://www.francesoir.fr/societe-sante/elisabeth-bik-la-deceleuse-de-fraudes-scientifiques-soupcons
32. Declaration of Helsinki – WMA – The World Medical Association [Internet]. [cited 2022 Dec 15]. Available from: https://www.wma.net/what-we-do/medical-ethics/declaration-of-helsinki/
33. Mehra MR, Desai SS, Kuy S, Henry TD, Patel AN. Retraction: Cardiovascular Disease, Drug Therapy, and Mortality in Covid-19. N Engl J Med. DOI: 10.1056/NEJMoa2007621. N Engl J Med. 2020 Jun 25;382(26):2582.
34. Roussel Y, Raoult D. Influence of conflicts of interest on public positions in the COVID-19 era, the case of Gilead Sciences. New Microbes and New Infections. 2020 Nov 1;38:100710.
35. Documents | Cour des comptes [Internet]. [cited 2023 Feb 23]. Available from: https://www.ccomptes.fr/fr/documents/62967
